# Exploring peer learning module vs. conventional tutorials: effects on engagement and learning outcomes among first-year medical students

**DOI:** 10.1186/s12909-024-06549-x

**Published:** 2025-01-21

**Authors:** Vinay Arasappa Vishwanath, Sindhu Raghuramaiah, Kavita Rasalkar

**Affiliations:** 1 Department of Physiology, Manipal Tata Medical College, Manipal Academy of Higher Education, Manipal, 576104 India; 2 Department of Biochemistry, Manipal Tata Medical College, Manipal Academy of Higher Education, Manipal, 576104 India

**Keywords:** Peer-learning, Conventional tutorial, Jigsaw technique, Active learning

## Abstract

**Background:**

In contemporary medical education, it is essential to raise student involvement and active participation in the learning process. By contrasting small-group peer learning modules with teacher-led conventional tutorial sessions, we aim to provide insights into their respective influences on learning outcomes and the overall learning experience among 150 first-year medical students.

**Methods:**

Each group consisted of 50 students. These students were further divided into two groups and a pretest was administered on the day of the session. One group engaged in conventional tutorials, while the other participated in a peer learning module. Post-tests and session feedback were provided after each session.

**Results:**

Results from the posttest revealed advancement in both learning approaches compared to the pretest. Compared to tutorials, the level of progress was much higher following peer learning with a p-value of < 0.05. Participants felt that while the tutorials helped them cover the full subject and saved time, they occasionally got monotonous and there was little active engagement. Students who participated in the peer learning method said that while interaction aided in a better learning experience, improved communication skills, and had more active participation, there was less time for discussion and some group members were reticent and ineffective in explaining the concepts.

**Conclusions:**

The peer learning module is thought to be superior to conventional tutorial classes since it promises active involvement from all students, promotes greater learning, and aids in skill improvement, thus assisting students to help each other in gaining insight into the process of active learning.

## Introduction

In the ever-evolving field of education, innovation is fueled by the search for efficient teaching strategies. Traditional didactic lectures and other teacher-driven learning activities may not ensure that every student effectively acquires knowledge [1, 2].

Conventional tutorials (CT) involve small groups of students and a tutor who interacts with the tutee to enhance the learning process and give greater attention to each individual student. This allows the tutees to engage with the tutors and get their questions answered [3]. The foundation of educational practices has historically been CT, but the emergence of small group Peer Learning Modules (PLM) offers an alternative paradigm that promotes interactive and collaborative learning environments.

PLM is a process of student centric learning strategies that enables students to learn from one another. This learning strategy can be facilitated by student seminars, problem or case-based learning which encourages students to actively explore literature, understand, analyze, share their insights, develop communication skills, promote self-confidence, and take up the onus of learning. Because they feel more at ease working with their peers, students are more likely to interact, ponder, and delve deeper into topics than they would in a classroom setting [4].

PLM is based on social constructivism model that encourages students to actively construct knowledge by interacting with peers, patients, and experienced healthcare professionals. Social engagement is crucial to help students develop clinical reasoning, communication skills, and professional identities [5].

A diverse array of teaching methods is used in medical education to help students gain the knowledge and abilities they need. These teaching strategies include didactic lectures, case studies, role-playing games, seminars, problem-based learning techniques, video demonstrations, etc [6].

An important focus of the recently adopted Competency Based Medical Education (CBME) curriculum by National Medical Commission is interactive teaching and learning approaches. It emphasizes the value of active involvement and participation in the learning process by stipulating that a significant portion (two-thirds) of the teaching schedules must be devoted to interactive sessions [7].

As a result, Peer learning is now an essential part of the curriculum in medical education as the emphasis moves from an educator-centered to a learner-centered paradigm. Peer learning can take various forms, such as peer tutoring, where one student teaches another in a one-on-one setting for mutual learning [8]. Alternatively, it can involve PLM, which facilitate a more structured approach to peer learning.

Jigsaw technique (JT) is one such PLM that has garnered increasing attention in medical education [1, 9]. This technique initially developed by Elliot Aronson in the 1970s, is a cooperative learning technique wherein students work together in small groups to master content and share their understanding with their peers [1, 10]. In the jigsaw method, each group member is given a particular piece of the learning material and it is the responsibility of each member to become an expert in that area. They then disseminate their knowledge to the remaining members of the group, making sure that everyone has a thorough comprehension of the subject at hand. As each participant bears responsibility for mastering their allocated segment and contributes to the group’s collective learning, this organized approach promotes accountability [11, 12]. Jigsaw technique is also known to enhance comprehension, promote collaboration, self-resilience, and foster critical thinking abilities in students [13].

A systemic review by Dornan T et al.; states that medical students learn best when they are active participants in the medical community, interacting with experienced practitioners in real-life contexts [14]. A study by Tait H et al. comparing CT to other teaching learning methods indicates that conventional tutorials offer significant benefits, such as personalized attention, enhanced student engagement, and better opportunities for in-depth discussion [15] When compared to other teaching techniques, Zhang H et al.‘s review and meta-analysis demonstrated the efficacy of peer learning in health professions education and its large impact on procedural skills development and equivalent effect on theoretical knowledge acquisition [16]. It was reported that postgraduate nursing students who use the peer learning technique in their biostatistics courses report lower exam anxiety and higher results [17]. A systematic review by Yu et al. observed that Peer-teaching had an influence on medical students’ objective learning outcomes that, in certain circumstances, seems to be comparable to conventional faculty-led tutorials [18].

The exact value of this peer learning over conventional teacher-led tutorial sessions is still up for debate. To assess the educational value of student- centric PLM like jigsaw technique and conventional teacher-centric tutorial sessions, we carried out a comparative analysis in this study. By comparing the impacts of conventional tutorial sessions with small-group peer learning on student engagement, information retention, critical thinking skills, and overall learning outcomes, this comparative analysis aims to close this gap in the literature. The purpose of this study is to give evidence-based insights that can improve the quality of medical education programs by carefully analyzing various teaching approaches using both quantitative and qualitative measurements.

## Materials and methods

**Fig. 1:**
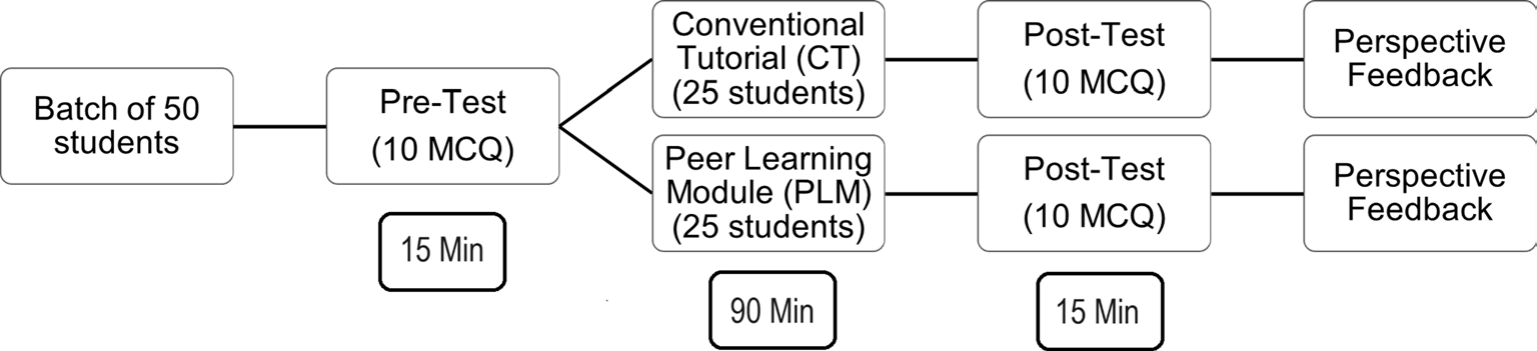
General study design of conventional tutorial (CT) and Peer learning module (PLM) that is jigsaw technique

The Study participants were 150 first year undergraduate medical students in a Private Medical college. Ethical clearance was obtained from the Institutional Human Ethics Committee, PES Institute of Medical Sciences and Research, Kuppam, Andhra Pradesh. 78 girls and 72 boys of I MBBS students participated in the study. 150 students were divided into batch A, B and C each comprising 50 students. The study was conducted for a period of one week. Batch B students attended on Monday and Thursday, batch C students on Tuesday and Friday, and Batch A students on Wednesday and Saturday. The topic for the tutorials for the first three days of the week was Physiology of Thyroid hormone and for the next three days of the week was Physiology of Glucocorticoids. The topic physiology of thyroid hormone and glucocorticoids were chosen for the study due to their similar concepts and level of difficulty. This information was conveyed to the students a week in advance. Students attend small group sessions for 2 h as per the schedule, a part of regular curriculum. Figure 1 illustrates the way the study was carried out according to the scheduled timetable for the entire six-day workweek.

The above cycle was repeated six days a week by shuffling the group according to the schedule to ensure that all the students had the opportunity to be a part of both PLM and CT. At the end of all the cycles, structured feedback was collected to assess the utility of the jigsaw technique in learning the concepts using a pre validated questionnaire from the study done by JR Dhage et al., (2017) [19] and perspective feedback was also obtained by all the participants for both teaching methods. The pre and post test includes the same 10 MCQ framed by the facilitators for that particular day, 2 MCQ from each of the subtopics were included to maintain the uniformity and degree of difficulty.

There were 25 students in CT, the faculty steered the session by explaining the topic to the students emphasizing the core concepts for 40 min. Students were engaged in question-and-answer sessions, where they responded to the questions posed by the faculty, also clarified their doubts regarding the topic for 40 min. The faculty summarized the topic of the day for 10 min.

**Fig. 2:**
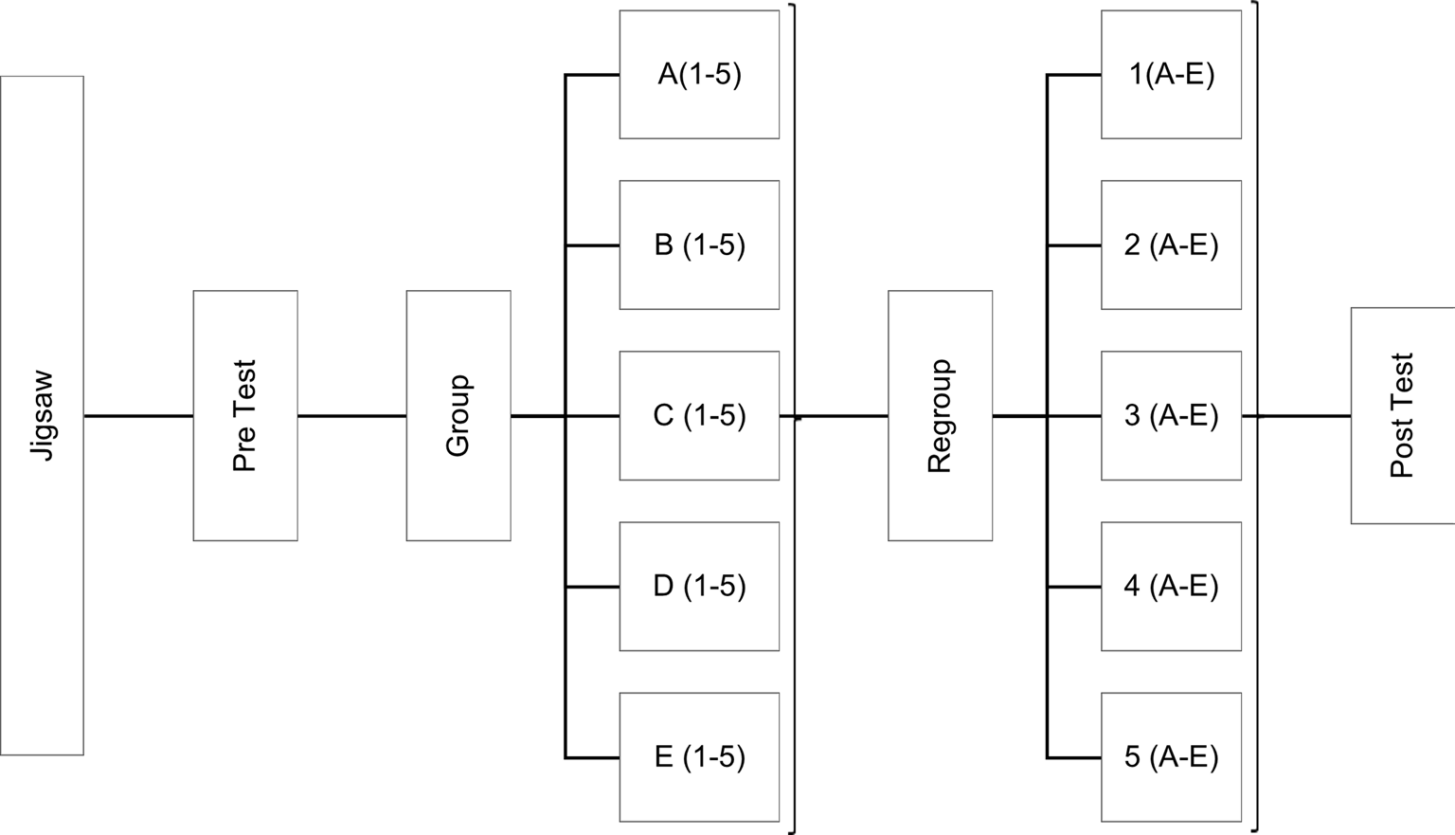
Jigsaw peer learning module designed for the study

There were 25 students in PLM. Figure 2 illustrates the concept of jigsaw technique used in our study. A faculty played the role of facilitator during this session. The students were given a handout of the subtopic of the day like functional anatomy of the gland/ synthesis of hormone/ mechanism of action/ actions of the hormone / applied physiology and were asked to read, discuss, and deliberate for 20 min in groups assigned as per Fig. 2. The students were asked to explain the concepts learnt in the first group to the rest of their peers in the second group after regrouping as in Fig. 2 for 50 min. Here each student got 8–9 min to explain the concept learnt to their peers. In the end, one student from each of the newly formed groups was randomly called upon by the facilitator to summarize the subtopic for 20 min.

### Statistical analysis

The student’s pre-and post-test results were tabulated into an excel spreadsheet. The pre and post test results did not follow the pattern of normal distribution, hence the scores were represented in the median and interquartile range, and a non-parametric test was employed to determine the significance after the Shapiro-Wilk test validated the data’s skewed distribution. The Wilcoxon Signed Rank Test was used to compare the students’ pre- and post-test results.

The students were further categorized into 4 groups based on the marks secured: low score, average score, high score, and cent score. To compare the pre-test results of the PLM and CT, as well as the post-test results of both, we performed a chi-squared test. A p-value below 0.05 was deemed statistically significant.

## Results

The pre and post test score was tabulated and Shapiro-wilk test predicted the P value of < 0.001 indicating the data is not normally distributed. Hence the pre and post-test score were expressed as median and interquartile range.

The median score secured by the students participating in conventional tutorials improved from 5 to 7 and the improvement was statistically significant with a p-value of < 0.001 (Table 1). The median score secured by the students participating in the Peer learning module improved from 5 to 8 and the improvement was statistically significant with a p-value of < 0.001 (Table 1). Both teaching-learning methods demonstrated a significant improvement in the post-test score but the improvement in the peer learning module was 10% higher than the conventional tutorial and is statistically significant with p value of < 0.001.

**Table 1:** Pre and post-test assessment of conventional tutorials and peer learning Module

			Median & Inter Quartile Range			Shapiro-Wilk		Wilcoxon Signed Rank Test
Assessment	TLM	*N*	25th	50th	75th	W	*P*	*P*- value
Pre-Test	Conventional Tutorial	150	3	5	7	0.95	< 0.001	< 0 0.001*
Post-Test	Conventional Tutorial	150	5	7	9	0.93	< 0.001	
Pre-Test	Peer Learning Module	150	3	5	7	0.95	< 0.001	< 0 0.001*
Post-Test	Peer Learning Module	150	6	8	10	0.9	< 0.001	

To assess the degree of impact in the pre and post-test score PLM and CT, the students were segregated into four categories based on their scores in pre and post-test. Poor performers being the one who scored between 0 and 3, average performers scored between 4 and 6, good performers scored between 7 and 9 and exceptional performers were the ones who secured 10 out of 10.

**Fig. 3:**
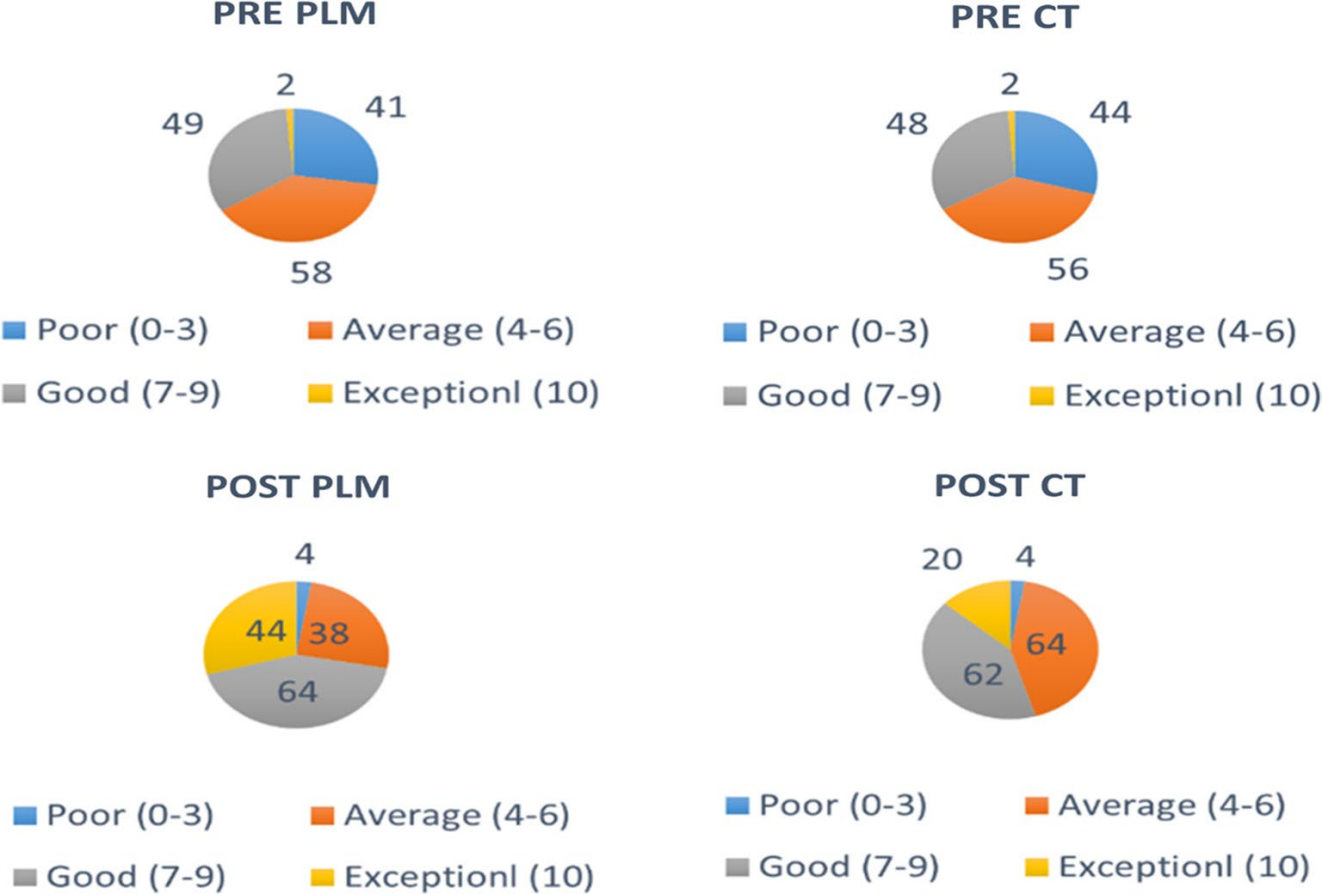
Pie chart depicting the performance of students in pre-test and post- test of PLM and CT

There was no statistically significant difference seen in the chi-squared test comparing number of students belonging to a specific category with respect to pretest results between PLM and CT (Table 2; Fig. 3). This suggests the two groups are nearly identical or similar.

**Table 2:** Represents the number of students belonging to each category based on their pre-test scores of PLM & CT

	Poor (0–3)	Average (4–6)	Good (7–9)	Exceptional (10)	X^2^	df	*P*-value
Pre PLM	41	58	49	2	0.151279	3	0.985
Pre CT	44	56	48	2			

When comparing the number of students in different categories based on post-test results between PLM and CT, the chi-squared test showed a statistically significant difference, with a p-value of 0.001 (Table 3; Fig. 3). Student test scores improved more after PLM than after CT, suggesting that a larger proportion of students who performed better belonged to the PLM-exposed group.

**Table 3:** Represents the number of students belonging to each category based on their post test scores of PLM & CT

	Poor (0–3)	Average (4–6)	Good (7–9)	Exceptional (10)	X^2^	df	*P*-value
Post PLM	4	38	64	44	15.6592	3	0.001*
Post CT	4	64	62	20			

**Fig. 4:**
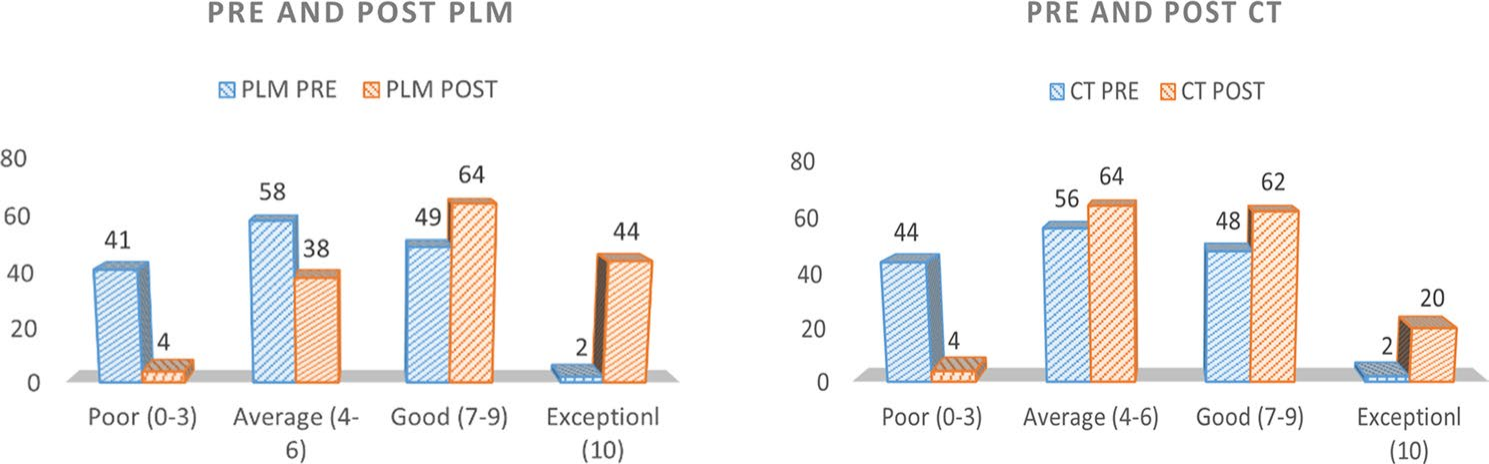
Bar chart representing number of students belonging to a particular category based on their Pre and Post test scores during PLM and CT

There were 41 and 44 low performers who secured 3 marks or less in the pre-test of PLM and CT respectively. The number of average performers was 58 and 56 in the pre-test of PLM and CT respectively. The number of good performers was 49 and 48 in the pre-test of PLM and CT respectively 2 students were exceptional performers securing 10 out of 10 the pre-test of both PLM and CT. (Fig. 4)

The number of low performers were reduced to 4 in the post-test of both PLM and CT. The number of average performers was 38 and 64 in the post-test of PLM and CT respectively. The number of good performers was 64 and 62 in the post-test of PLM and CT respectively. The number of exceptional performers increased from 2 during the pre-test of PLM and CT to 44 and 20 in the post test of PLM and CT respectively (Fig. 4).

93% of students rated JT as good (45%) and excellent (48%). Moreover, 89% of students expressed a preference for JT over conventional tutorials. Additionally, 84% of students opined that every step of this activity was important. Furthermore, 86% of participants said they were continuously motivated during the sessions. 89% of them thought that they developed some skills or enhanced the skills that they already possessed and 85% of the students believed that learning objectives were satisfactorily met. 94% of the students expressed that they had a clear understanding of the topic by JT. The students reported substantial benefits in various aspects, including the realization of the importance of participation (89%), proper planning/strategy (87%), problem-solving skills (85%), creativity/resources (83%), teamwork (79%), time management (77%), and leadership (73%). 94% of the students endorsed the JT to be implemented regularly (Table:4).

**Table 4:** Structured feedback of students regarding jigsaw technique

Sl No	Feedback Questions		Rating in Likert scale given by 150 students for Jigsaw learning activity in percentage of students			
			Poor (1)	Average (2)	Good (3)	Excellent (4)
1	Rate the Jigsaw active learning activity		1	6	45	48
2	Rate this learning style over traditional teaching style		2	8	49	40
3	Do you think each step in this activity is important?		2	15	45	39
4	Does this activity motivate to study the entire topic in detail? Rate it accordingly.		3	10	41	45
5	Have you developed some skills or enhanced skills you already possessed due to this activity? Rate it accordingly		2	9	48	41
6	Rate this activity in terms of fulfilment of learning objectives		2	12	48	37
7	Rate this activity according to the understanding of topics were developed.		1	5	49	45
8	Rate this activity in a way it helped you to realize the importance of these skills	Participation	0	11	45	44
9		Leadership	6	22	48	25
10		Teamwork	2	19	34	45
11		Creativity / resources	2	15	43	40
12		Relevancy to topic	2	14	49	35
13		Problem solving skill	2	13	52	33
14		Time management	3	20	43	34
15		Proper planning /strategy	2	11	48	39
16	Rate this activity good enough to perform on regular basis.		2	4	46	48

The exploratory factor analysis of the structured feedback questionnaire was undertaken, items 1, 2,3,4,8,14 assessed the impact on motivation, the items 5,6, 9,10,11,12,13 assessed the impact on skill development and the item 7 assess the student engagement, it reveals strong correlation between JT on these parameters. (Table:5)

**Table 5:** Exploratory factor analysis of the structured feedback questionnaire

Sl No	Survey Item	Motivation	Skills Development	Engagement
1	Rate the Jigsaw active learning activity	0.83	-	-
2	Rate this learning style over traditional teaching style	0.67	-	-
3	Do you think each step in this activity is important?	0.62	-	-
4	Does this activity motivate to study the entire topic in detail?	0.88	-	-
5	Have you developed some skills or enhanced skills…	-	0.82	-
6	Rate this activity in terms of fulfilment of learning objectives	-	0.75	-
7	Rate this activity according to the understanding of topics	-	-	0.86
8	Rate this activity in a way it helped you to realize the importance of these skills: Participation	0.81	-	-
9	Rate this activity in a way it helped you to realize the importance of these skills: Leadership	-	0.74	-
10	Rate this activity in a way it helped you to realize the importance of these skills: Teamwork	-	0.73	-
11	Rate this activity according to Problem Solving Skills	-	0.76	-
12	Rate this activity in a way it helped you to realize the importance of these skills: Time Management	-	0.68	-
13	Rate this activity in a way it helped you to realize the importance of these skills: Proper Planning/Strategy	-	0.77	-
14	Rate this activity good enough to perform on a regular basis.	0.75	-	-

PLM and CT have their own share of strengths and weakness with respect to students’ perception, and the perspective feedback received has been tabulated (Table 6). The comprehensive analysis of the feedback revealed PLM fosters engagement, collaboration, and soft skills but requires better facilitation and time management. Structured guidance and contingency plans could mitigate the challenges of incomplete topic coverage. While conventional tutorials ensure topic coverage and are time-efficient, they lack engagement and fail to promote active learning or a supportive environment for student participation.

**Table 6:** Perspective feedback of students on peer learning Module & conventional tutorials and

Teaching Learning Methods	Positive feedback	Negative feedback
Peer learning Module	• The interactions were good. • Was active throughout the session. • Able to improve my communication skills. • Was able to learn something which I would have missed when I read by myself.	• Time for discussion was less. • Some group members are hesitant and not effective in explaining. • If a student fails to explain, other members tend to lose that part of topic.
Conventional Tutorial	• Time saving • Helps to cover entire topic	• Boring & not interesting • Fear of being judged by my friends. • No active participation from the students

## Discussion

In contemporary higher education, there has been a notable shift towards prioritizing teaching and learning methodologies. The teaching methods play a pivotal role in the lives of medical students for them to be good professionals [20]. There has been a discernible shift in the educational landscape in recent years from the conventional understanding of the teacher as the principal expert, using mostly didactic approaches, to a more modern understanding of the teacher as a learning facilitator. [21] In this changing paradigm, teachers are no longer solely responsible for being the exclusive purveyors of information; instead, they are expected to assist students in identifying resources and paths toward knowledge [20]. 

Our study showed that the conventional, as well as the newer peer learning module using JT, are effective in improving their performance. It was also found that the performance of the students was much better after the PLM in comparison with the conventional tutorials (Table 1). A study by Sharma S et al., (2019) and a study by Verma SR et al., (2017) support our results which showed an improvement in the performance of the students following JT. [22, 23] On contrary to our findings, some of the studies reported that there were no significant changes in posttest following both the conventional tutorials and JT.[24, 25].

When comparing the categorized pretest marks between the PLM and CT, the chi-squared test showed no statistically significant difference, according to this study. Implying that pretest marks secured by the students are almost similar, emphasizing the topics involved in assessing the teaching learning methods are of similar degree of difficulty. A study by Pai KM et al.,(2014) suggests a similar finding in their study which revealed that there was no discernible difference between the two groups of students with respect to their performance in pre- test [26]. Our study also noted that a significant difference (p-value of 0.001) was found in comparing the categorized marks that students received in the post-test between PLM and CT, demonstrating a markedly increase in students’ performance in post-test score after PLM as opposed to CT. Of the 150 students who completed the pretest, 41 were low performers in the PLM test and 44 were low performers in the CT pretest. Furthermore, two students were exceptional performers in both PLM and CT pretests with the score 10 out of 10. Following PLM and CT, student post-test results improved; out of 150, the low performers were only four. In both PLM & CT group. the number of exceptional performers increased to 44 and 20 in the post test in PLM and CT group respectively.

Perspective feedback of students for the PLM in our study revealed that majority of the students (93%) opined that this newer PLM using JT was highly favorable (Table 4). Moreover, many students expressed a preference for PLM over conventional tutorials. A study by Goolsarran N et al., (2020) suggested that the students felt that participating in JT improved their medical knowledge and enhanced learner satisfaction in comparison with the traditional tutorials which was in alignment with our findings [27]. In the present study, most of the students pronounced that every phase of the approach was crucial, highlighting the usefulness of PLM in supporting teaching and learning activities. Most of them said they were incessantly motivated during the sessions, underscoring JT’s compelling quality. Similar to this finding, after employing the JT, Sanaie et al., [28] saw a significant improvement in students’ self-regulated learning abilities and academic motivation [28]. The current study showed that majority of the students expressed that they developed essential extra skills and /or enhanced the skills that they already possessed, and they also believed that learning objectives were satisfactorily met. Notably, most of them claimed to have a high sense of understanding and mastery of the material, which they attributed to their involvement in Peer learning sessions. Several studies are also in agreement with our study findings that revealed better understanding, learning and knowledge retention by JT [12, 29, 30]. Additionally, in our study, students acknowledged the myriad advantages associated with JT, including how it promotes involvement, strategic planning, problem-solving skills, ingenuity, teamwork, time management, and leadership qualities. Similar findings were noted in a few studies wherein this teaching strategy of PLM using JT helped students become more self-assured [31], improved their communication skills, encouraged peer support, developed logical thinking, sharpened their problem-solving abilities, increased motivation [32], and stimulated critical thinking [31]. A study by Jeppu AK et al., [12] specified that the use of outcome-based learning environments to promote mastery of core academic subjects and the development of critical thinking, cooperation, creativity, and other vital abilities is essential for medical students [12]. In the current study, the students also believed that the PLM using JT needs to be implemented regularly (Table 4).

The Exploratory factor analysis of the feedback questionnaire reveals Jigsaw learning approach has influence on students’ engagement, skill development, and motivation. Each component is a crucial component of the educational experience for the students and offers insightful information for the next instructional plans (Table 5).

The Jigsaw exercise has shown a strong correlation with increased student motivation, according to their feedback. This result is consistent with previous research that indicates collaborative learning settings might improve intrinsic motivation by encouraging peer responsibility and active engagement [33]. Motivation is emphasized heavily because motivated students are more likely to put in the time and effort necessary for studying, which improves academic performance.

Students reported that the Jigsaw method helped them improve key competencies, contributing significantly to their skills development. High loadings on items related to participation, leadership, teamwork, and problem-solving suggest that students not only perceived skill development as a direct outcome of their participation but also recognized its broader relevance in their future careers. This finding is particularly significant in the context of medical and nursing education, where teamwork and communication skills are integral to effective clinical practice [34]. 

Engagement captures aspects related to students’ involvement and understanding of the topics covered. Engaged learners are more likely to retain information and develop a deeper understanding of complex subjects, which is vital in the medical field where comprehensive knowledge is essential [35]. The Jigsaw method encourages students to take ownership of their learning, fostering a sense of agency that is often lacking in traditional lecture-based formats.

Students offered a variety of perspectives on conventional tutorials, stressing both benefits and issues. They valued the ability to cover the complete subject in the conventional instructional format as well as the time-saving element. They were unhappy with what they considered it to be monotonous, calling it “*boring*” and “*not interesting*.” A study by Pahwa et al., (2022) revealed a similar finding in which the students felt that the traditional method of teaching made them passive [36]. Participants also expressed similar concerns about being assessed by others and a perceived lack of active participation from students in our study which echoes with other studies [36]. These revelations highlight how crucial it is to consider the opinions and experiences of students while assessing and improving medical education teaching strategies.

Students also gave feedback regarding the peer learning JT. They thought that the environment was conducive to collaborative learning as they stated, “*Interactions were good*”. They also felt the active involvement and participation as they quoted, “*Was active throughout the session*”. Additionally, students indicated a perceived improvement in this area by highlighting the positive effects of the peer learning module on their communication skills as they stated, “*Able to improve my communication skills*”. A study by Tran VD et al., (2012) showed similar opinions of students for JT in which students felt that their communication skills improved and experienced active participation [1, 37]. Participants recognized the importance of group discussions in promoting learning, pointing out that they learned something from the discussions that they might not have learned from solitary study as they asserted, “*Was able to learn something which I would have missed when I read by myself*”.

Participants did, however, also point out areas that needed improvement. The apparent lack of time allotted for discussion was one issue that was raised, indicating the need for longer sessions or improved time management techniques to enable more in-depth topic analysis. Students also mentioned, “*Some group members are hesitant and not effective in explaining*” this showed that here have also been issues with some group members’ ability to effectively convey concepts, which could leave the rest of the group with knowledge gaps. “*If a student fails to explain*,* other members tend to lose that part of topic*.” Participants also highlighted the interconnectedness of collaborative learning by expressing concern that other group members may find it difficult to understand a particular aspect of the material if a student fails to present it well. The incomplete participation of all group members or the poor performance of some individuals, which subsequently affects the group’s success, is another problem that these studies draw attention to [1, 38, 39]. This disadvantage can be overcome by a trained facilitator helping the respective group.

The effectiveness of conventional tutorials compared to peer learning modules in enhancing student learning experiences was explored in this study. Though they were deemed boring with little active participation, tutorials were found to effectively cover concepts and save time. On the other hand, peer learning modules promoted active participation, interaction, and communication skills but had drawbacks including little time for debate and differing degrees of efficacy among group members. This emphasizes how crucial it is to strike a balance between engagement and efficiency to maximize learning.

### Limitations of the study

The data for this study was gathered just at one institution, limiting the applicability of the findings to other places and settings. The research depended on self-reported data, which is susceptible to biases due to personal preference and type of learner. Moreover, the questionnaire format may not have thoroughly encapsulated the diverse experiences and viewpoints of the students about PLM & CT.

## Conclusion

This study shows that the students performed well following both the conventional tutorials and peer learning module using jigsaw technique, although most of the students preferred PLM. The findings suggest that small-group peer learning stands out as a promising strategy, greatly increasing student participation and enabling a deeper comprehension of the material. In addition to developing vital abilities like teamwork, communication, and critical thinking, peer learning creates a safe space where students may help and learn from one another. On the other hand, while still beneficial for offering thorough treatment of subjects, traditional tutorial sessions do not have the same interactive and collaborative components as peer learning environments.

We recommend considering the viewpoints and experiences of students when developing and putting into practice instructional strategies in medical education. To ensure the comprehensive growth of upcoming healthcare professionals and to continually improve medical education practices, further investigation, and study into the best ways to combine peer learning approaches with traditional teaching methods is necessary.

## Data Availability

The data that support the findings of this study are available from the corresponding author upon reasonable request.

## Abbreviations

CBME: Competency Based Medical Education
CT: Conventional Tutorial
JT: Jig-Saw Technique
PLM: Peer Learning Module
TLM: Teaching Learning Methods

## References

[1] Lalit M, Piplani S. Assessing the outcome of implementation of jigsaw technique as a learning tool and its effect on performance of 1st year medical students in anatomy. Natl J Clin Anat. 2021;10(2):97–102. 10.4103/NJCA.NJCA_57_20.

[2] Jafariyan M, Matlabi M, Esmaeili R, Kianmehr M. Effectiveness of teaching: jigsaw technique vs lecture for medical students’ physics course. Bali Med J. 2017;6(3):529–33. 10.15562/bmj.v6i3.400.

[3] Balwant PT, Doon R. Alternatives to the conventional ‘Oxford’ tutorial model: a scoping review. Int J Educational Technol High Educ. 2021;18(1):29. 10.1186/s41239-021-00265-y.

[4] Biggs JB, Tang CS, Society for Research into Higher Education). (with. (2011). *Teaching for quality learning at university: What the student does* (4th edition). McGraw-Hill, Society for Research into Higher Education & Open University Press.

[5] Cho H, Jeong H, Yu J, Lee J, Jung HJ. Becoming a doctor: using social constructivism and situated learning to understand the clinical clerkship experiences of undergraduate medical students. BMC Med Educ. 2024;24(1):236. 10.1186/s12909-024-05113-x. 38443907 10.1186/s12909-024-05113-x PMC10916183

[6] Jana PK, Sarkar TK, Adhikari M, Chellaiyan VG, Ali FL, Chowdhuri S. A study on the preference of teaching methods among medical undergraduate students in a tertiary care teaching hospital, India. J Educ Health Promotion. 2020;9. 10.4103/jehp.jehp_232_20. 10.4103/jehp.jehp_232_20 PMC7709748 33282980

[7] Rustagi SM, Verma N, Prakash S, Dave V, Dhuria R. Perception analysis of students and faculty of a recently implemented interactive teaching session in anatomy using ‘Jigsaw Technique’in a north Indian medical college. J Educ Technol Health Sci. 2020;7(1):17–22. 10.18231/j.jeths.2020.004.

[8] Moore-West M, Hennessy SA, Meilman PW, O’Donnell JF. The presence of student-based peer advising, peer tutoring, and performance evaluation programs among US medical schools. Acad Med. 1990;65(10):660–1. 10.1097/00001888-199010000-00018. 2261049 10.1097/00001888-199010000-00018

[9] Slavin RE. Instruction based on cooperative learning. Handb Res Learn Instruction. 2011;4(2):12–23.

[10] Gillies RM. Teachers’ and students’ verbal behaviours during cooperative and small-group learning. Br J Educ Psychol. 2006;76(2):271–87. 10.1348/000709905X52337. 16719964 10.1348/000709905X52337

[11] Yang X. (2023). A Historical Review of Collaborative Learning and Cooperative Learning. TechTrends, 1–11. 10.1007/s11528-022-00823-9 10.1007/s11528-022-00823-9 PMC9860218 36711122

[12] Jeppu AK, Kumar KA, Sethi A. We work together as a group’: implications of jigsaw cooperative learning. BMC Med Educ. 2023;23(1):734. 10.1186/s12909-023-04734-y. 37803418 10.1186/s12909-023-04734-y PMC10559587

[13] Krych AJ, March CN, Bryan RE, Peake BJ, Pawlina W, Carmichael SW. Reciprocal peer teaching: students teaching students in the gross anatomy laboratory. Clin Anatomy: Official J Am Association Clin Anatomists Br Association Clin Anatomists. 2005;18(4):296–301. 10.1002/ca.20090. 10.1002/ca.20090 15832347

[14] Dornan T, Littlewood S, Margolis SA, Scherpbier A, Spencer J, Ypinazar V. How can experience in clinical and community settings contribute to early medical education? A BEME systematic review. Med Teach. 2006;28(1):3–18. 10.1080/01421590500410971. 16627313 10.1080/01421590500410971

[15] Tait H, Godfrey H. Defining and assessing competence: an interview study with stakeholders in education and industry. Educ Train. 1999;41(7):341–9.

[16] Zhang H, Liao AWX, Goh SH, Wu XV, Yoong SQ. Effectiveness of peer teaching in health professions education: a systematic review and meta-analysis. Nurse Educ Today. 2022;118:105499. 10.1016/j.nedt.2022.105499. 35961134 10.1016/j.nedt.2022.105499

[17] Babaahmadi A, Maraghi E, Moradi S, Younespour S. Comparison between peer learning and conventional methods in Biostatistics Course among postgraduate nursing students’ final score, statistics and test anxiety: a quasi-experimental study with a Control Group. Shiraz E-Medical J. 2021;22(11). 10.5812/semj.111984. Article 11.

[18] Yu T-C, Wilson NC, Singh PP, Lemanu DP, Hawken SJ, Hill AG. Medical students-as-teachers: a systematic review of peer-assisted teaching during medical school. Adv Med Educ Pract. 2011;2:157–72. 10.2147/AMEP.S14383. 23745087 10.2147/AMEP.S14383 PMC3661256

[19] Dhage JR, Patil MS, Pawar AB. Implementation and feedback analysis of jigsaw active learning method. J Eng Educ Transformations. 2017;30(3):192–9.

[20] Kumar CSV, Kalasuramath S, Reddy SJ, Reddy RSN. Jigsaw: a step toward co-operative learning among medical and nursing students. Archives Med Health Sci. 2023;11(1):25–31. 10.4103/amhs.amhs_1_23.

[21] Slavin RE. Research on cooperative learning and achievement: what we know, what we need to know. Contemp Educ Psychol. 1996;21(1):43–69. 10.1006/ceps.1996.0004.

[22] Sharma S, Chauhan S, Kaur M. Introduction and assessment of jigsaw method of teaching on challenging topics in physiology for first year medical students. Int J Physiol. 2019;7(4):238–45. 10.37506/ijop.v7i4.99.

[23] Srikanth Varma R. Jigsaw method as a teaching methodology in orthopaedic clinical examination: a study conducted on 8th semester MBBS students in KAMSRC. J Educational Res Med Teacher. 2017;5(1):23–5.

[24] Puppalwar PV, Jambhulkar RK. Jigsaw technique-A novel method of teaching biochemistry to medical undergraduates. Int J Med Sci Public Health. 2019;8(12):1052–6. 10.5455/ijmsph.2019.0823204102019.

[25] Sagsoz O, Karatas O, Turel V, Yildiz M, Kaya E. Effectiveness of jigsaw learning compared to lecture-based learning in dental education. Eur J Dent Educ. 2017;21(1):28–32. 10.1111/eje.12174. 10.1111/eje.12174 26547392

[26] Pai KM, Rao KR, Punja D, Kamath A. The effectiveness of self-directed learning (SDL) for teaching physiology to first-year medical students. Australasian Med J. 2014;7(11):448–53. 10.4066/AMJ.2014.2211. 10.4066/AMJ.2014.2211 PMC4259209 25550716

[27] Goolsarran N, Hamo CE, Lu WH. Using the jigsaw technique to teach patient safety. Med Educ Online. 2020;25(1):1710325. 10.1080/10872981.2019.1710325. 31884898 10.1080/10872981.2019.1710325 PMC6968255

[28] Sanaie N, Vasli P, Sedighi L, Sadeghi B. Comparing the effect of lecture and jigsaw teaching strategies on the nursing students’ self-regulated learning and academic motivation: a quasi-experimental study. Nurse Educ Today. 2019;79:35–40. 10.1016/j.nedt.2019.05.022. 31102795 10.1016/j.nedt.2019.05.022

[29] Nusrath A, Dhananjaya SY, Dyavegowda N, Arasegowda R, Ningappa A, Begum R. Jigsaw Classroom: Is it an Effective Method of Teaching and Learning? Student’s Opinions and Experience. J Clin Diagn Res. 2019;13(2). 10.7860/JCDR/2019/39613.12540.

[30] Uppal V, Uppal N. Flipped jigsaw activity as a small group peer-assisted teaching learning tool in Biochemistry Department among Indian Medical Graduate: an experimental study. Biochem Mol Biol Educ. 2020;48(4):337–43. 10.1002/bmb.21355. 32429002 10.1002/bmb.21355

[31] Raoufi S, Farhadi A, Sheikhian A. Impact of the team effectiveness design of teaching on critical thinking, self-confidence, and learning of nursing students. J Med Educ. 2014;9(2):23–32.

[32] Haghani F, Rahimi M, Ehsanpour S. An investigation of perceived feedback in clinical education of midwifery students in Isfahan University of Medical Sciences. Iran J Med Educ. 2014;14(7):571–80. http://ijme.mui.ac.ir/article-1-3164-en.html.

[33] Johnson DW, Johnson RT. Cooperative learning: improving university instruction by basing practice on validated theory. J Excellence Coll Teach. 2017;28(3):1–27.

[34] Levine DR, O’Brien AM, Melton S. The role of teamwork and communication skills in healthcare delivery: a study of interdisciplinary education and practice. J Interprof Care. 2018;32(2):145–53. 10.1080/13561820.2017.1398126.

[35] Fredricks JA, Blumenfeld PC, Paris AH. School engagement: potential of the concept, state of the evidence. Rev Educ Res. 2004;74(1):59–109. 10.3102/00346543074001059.

[36] Pahwa AR, Dudani S, Gangadharan V, Gulati R. Introduction of the jigsaw technique of cooperative learning in teaching pathology to medical undergraduates. CHRISMED J Health Res. 2022;9:252. 10.4103/cjhr.cjhr_19_22.

[37] Tran VD, Lewis R. The effects of Jigsaw Learning on Students’ attitudes in a Vietnamese Higher Education Classroom. Int J High Educ. 2012;1(2):9–20. 10.5430/ijhe.v1n2p9.

[38] Persky AM, Pollack GM. A hybrid jigsaw approach to teaching renal clearance concepts. Am J Pharm Educ. 2009;73(3). 10.5688/aj730349. 10.5688/aj730349 PMC2703274 19564992

[39] Kumar VCS, Kalasuramath S, Patil S, Kumar RKG, Taj SKR, Jayasimha VL, Chacko T. Effect of jigsaw co-operative learning method in improving cognitive skills among medical students. Int J Curr Microbiol Appl Sci. 2017;6(3):164–73. 10.20546/ijcmas.2017.603.018.

